# Enhanced U-Net++ for Improved Semantic Segmentation in Landslide Detection

**DOI:** 10.3390/s25092670

**Published:** 2025-04-23

**Authors:** Meng Tang, Yuelin He, Muhammed Aslam, Edore Akpokodje, Syeda Fizzah Jilani

**Affiliations:** 1 Department of Computer Science, Aberystwyth University, Penglais, Aberystwyth SY23 3DB, UK; met57@aber.ac.uk (M.T.); yuh22@aber.ac.uk (Y.H.); eta@aber.ac.uk (E.A.); 2 Department of Physics, Aberystwyth University, Penglais, Aberystwyth SY23 3DB, UK; sfj7@aber.ac.uk

**Keywords:** landslide detection, semantic segmentation, U-Net++, attention mechanisms, disaster risk assessment

## Abstract

Landslide detection and segmentation are critical for disaster risk assessment and management. However, achieving accurate segmentation remains challenging due to the complex nature of landslide terrains and the limited availability of high-quality labeled datasets. This paper proposes an enhanced U-Net++ model for semantic segmentation of landslides in the Wenchuan region using the CAS Landslide Dataset. The proposed model integrates multi-scale feature extraction and attention mechanisms to enhance segmentation accuracy and robustness. The experimental results demonstrate that ASK-UNet++ outperforms traditional methods, achieving a mean intersection over union (mIoU) of 97.53%, a Dice coefficient of 98.27%, and an overall accuracy of 96.04%. These findings highlight the potential of the proposed approach for improving landslide monitoring and disaster response strategies.

## 1. Introduction

A landslide is a geological phenomenon in which the rock and soil mass of a mountain slide down along a certain weak surface as a whole or in a dispersed manner due to natural or human factors [1]. The main types include rockslides, soil landslides, debris slides, and collapses. Rockslides occur when the original mechanical equilibrium of rock slopes is disrupted by factors such as gravity, rainfall, earthquakes, and human engineering activities. In these cases, the rock mass slides downwards along its internal weak structural planes, such as joints and faults, due to the sliding force exceeding the anti-sliding force, forming a landslide as a whole [2]. Similarly, soil landslides are caused by a decrease in soil shear strength or an increase in sliding force, which disrupts the original mechanical equilibrium. If affected by rainfall, earthquakes, river erosion, human loading, etc., the soil will slide downwards along the weak surface or weak zone [3]. Debris slides occur primarily in mountain valleys, where the presence of abundant loose solid materials, such as weathered rock, soil, and mine tailings, serves as a prerequisite for their formation. Steep terrain, large valley slopes, and abundant short-term water sources such as rainfall, ice melt, and snowmelt contribute to debris slide formation. Collapse often occurs on high and steep slopes due to weathering causing rock fragmentation on the mountain surface, earthquake, blasting, and other shocks, as well as rainstorm scouring and river erosion at the foot of the slope, resulting in sudden collapse of local rock or soil mass of the mountain.

### 1.1. Global Impact of Landslides

Landslides occur frequently worldwide, bringing serious disasters to many countries and regions. The 2008 Wenchuan earthquake triggered a large number of landslides. Earthquakes generate strong vibrations, causing damage to the internal structure of mountains, loss of stability of rock and soil masses, and rapid sliding of a large amount of soil and rock along slopes. Many residential houses collapsed due to the inability to withstand the impact of landslides, resulting in a large number of casualties. Industrial factories, commercial facilities, and other infrastructure were buried or destroyed by landslides, leading to substantial material damage and direct economic losses. In terms of transportation infrastructure, roads have been buried by soil and rocks, with a total length of hundreds of kilometers. Bridges were broken due to landslide impacts, resulting in a complete paralysis of transportation. It was difficult for rescue vehicles and supplies to be quickly transported to the affected areas, greatly hindering rescue and reconstruction work [4]. Due to complex geological structures and concentrated rainfall, landslides occur frequently in the Himalayan region of India [5]. When a landslide occurs, a large number of houses located at the foot of the mountain or near the slope are knocked over and buried by the landslide body, and many residents lose their homes, posing a serious threat to their safety. Large areas of farmland can be covered with soil and rocks caused by landslides, resulting in crops being unable to continue growing and agricultural irrigation facilities being damaged. Subsequent agricultural production is difficult to carry out normally, leading to a significant reduction in grain production and seriously affecting the local agriculture-dominated economic structure [6]. California is located at the junction of tectonic plates in the United States, and frequent seismic activity has led to landslides. Landslides have caused damage to a large number of residential buildings, forcing many residents to evacuate their homes urgently and completely disrupting their normal living order. Roads have collapsed and been buried due to landslides, causing chaos in the transportation network. Commercial activities that rely on transportation, such as logistics, have been forced to come to a halt, resulting in increased operating costs for businesses. Some companies have even stopped production due to the inability to transport raw materials and products, seriously hindering local economic development [7].

### 1.2. Methods for Landslide Investigation

Assessing landslide risk requires a combination of investigation methods to ensure accuracy and reliability. Common approaches include ground investigation, geological exploration, and remote sensing techniques, each providing unique insights into landslide assessment. Ground investigations rely on the professional knowledge and experience of investigators, who conduct on-site assessments directly in landslide-prone areas. By means of visual observation, measurement, and other methods, information on the morphology, rock and soil characteristics, and surrounding environment of landslide bodies can be obtained to determine the possibility and development status of landslides [8]. Investigators visit potential landslide sites and carefully observe the terrain and landforms, checking for abnormal slope shapes, cracks, collapses, and other warning signs. A geological compass is used to measure the orientation of rock layers, including their strike, dip, and dip angle, to assess stability. Additionally, tools such as a tape measure and total station are employed to determine the geometric parameters of the landslide body, including its boundaries, length, width, and height. Vegetation growth patterns are also examined, as anomalies such as skewed trees and exposed roots may indicate subsurface displacement of rock and soil. Furthermore, interviews with local residents provide historical insights into past landslide events and related occurrences. In addition to ground investigations, geological exploration offers deeper insights into the internal composition and structure of landslide-prone areas by directly obtaining rock and soil samples through methods such as drilling and pit excavation. These samples are subsequently analyzed in the laboratory to determine their physical and mechanical properties, providing essential data for assessing landslide risk [9]. During drilling, specialized equipment is used, such as a rotary drilling rig, to drill the drill rod and drill bit deep underground. During the drilling process, rock core samples are collected at different depths. The rock cores are observed and analyzed in the laboratory, considering their lithology, structure, and other characteristics, and parameters such as the density, moisture content, porosity, and shear strength of the rock and soil mass are measured. Pit exploration is the process of digging exploration pits at selected locations, typically at shallow depths. The layering of soil layers and the development of rock joints and fissures on the pit wall is directly observed, their spacing and orientation measured, geological sketches drawn, the underground geological structure intuitively understood, and the location and characteristics of potential sliding surfaces determined. To complement these ground-based methods and achieve broader spatial coverage, remote sensing techniques are used to detect landslides based on the unique reflection and radiation properties of electromagnetic waves from different land surfaces. Remote sensing equipment detects landslides by receiving these electromagnetic wave signals, identifying information such as terrain type, shape, and range [10]. Satellites or aircraft equipped with optical and radar sensors collect imagery over target areas. In images obtained by optical remote sensing, the color tone and texture of landslide bodies are different from those of surrounding normal areas, which can be used to identify the scope and shape of a landslide. Synthetic-aperture radar (SAR) uses interferometric measurement technology to obtain information on small surface deformations and monitor potential landslide areas by transmitting and receiving radar waves. After obtaining remote sensing images, preprocessing such as radiometric correction and geometric correction is performed to remove noise and errors. Then, image classification algorithms are used, such as supervised classification and unsupervised classification, to classify the ground objects in the image and extract landslide information. Finally, geographic information system (GIS) technology, spatial analysis, statistics, and visualization displays of landslide data are combined to provide support for landslide monitoring and early warning systems. In addition to optical remote sensing, radar-based techniques, particularly interferometric synthetic-aperture radar (InSAR), have become indispensable for detecting subtle ground deformations associated with landslides. InSAR technology utilizes radar signals to detect surface deformations that occur during landslide events. When radar waves are emitted and received at different time intervals, changes in the land surface cause corresponding changes in the radar signal phase. By analyzing these phase differences, researchers can calculate displacement, velocity, and other critical information about landslide movements. The process involves acquiring multi-temporal radar image data from satellites, followed by preprocessing steps including radiometric correction and denoising to enhance data quality. The interferometric processing then generates interferograms, from which terrain and deformation information is extracted through phase unwrapping. By incorporating digital elevation models (DEMs) to remove terrain phase influences, accurate surface deformation measurements can be obtained. Recent advancements in the InSAR methodology, such as the sequential turning point detection (STPD) technique, have further improved the ability to estimate landslide reactivation times with greater precision [11]. In another study, the SBAS (Small Baseline Subset) approach was used with Sentinel-1 radar data to monitor ground deformation on Aegina Island, demonstrating the reliability of InSAR for detecting active geological phenomena [12]. Continuous improvements in spaceborne InSAR technology have significantly enhanced both landslide monitoring capabilities and susceptibility mapping, making it an essential tool in modern landslide risk assessment frameworks [13]. To comprehensively evaluate landslide risks, researchers integrate different methods, each offering unique insights. As summarized in Table 1, the combination of these techniques provides a well-rounded approach, balancing their individual strengths and limitations.

### 1.3. Specific Challenges Faced by Current Research Methods

In the study of landslides, remote sensing technology is an important technique, but it also faces many challenges, mainly reflected in geographical complexity and data scarcity and annotation difficulties. In terms of geographical complexity, the terrain in mountainous areas is extremely complex. Many mountainous areas have extremely undulating terrain, such as the southwestern region of China, where high mountains and canyons alternate and the height difference can reach thousands of meters. This type of terrain can cause severe terrain shadows, and in remote sensing images, the information in the shadow areas may be missing or obscured, making it difficult to accurately capture landslide information. Moreover, the reflection and scattering of remote sensing signals vary depending on the geological structure and rock type. For example, in granite and shale areas there are significant differences in signal characteristics, which makes landslide features manifest differently in different geological backgrounds, increasing the difficulty of interpretation. In addition, vegetation coverage also causes serious interference, and dense vegetation may completely cover the landslide body, making it difficult for remote sensing images to present the real landslide situation. A scarcity of data and difficulties in annotation also plague research. Obtaining long-term, high-resolution remote sensing data is not easy, and the limited revisit period of satellites makes it difficult to capture dynamic changes of landslides in a timely manner. And high-resolution data are expensive, and data processing and storage also require a large amount of resources. In the labeling process, accurately labeling landslide areas requires professional knowledge, and field research is limited by terrain, weather, and other conditions, that make it difficult to reach many remote mountainous areas. Moreover, due to different knowledge backgrounds and the experience level of different annotators, there are differences in annotation results, which affect the accuracy and consistency of the data, and thus affect the accuracy of the models established based on these data [19]. This research presents a novel deep learning-based approach to landslide semantic segmentation, addressing the limitations of traditional methods that often require extensive expert intervention and manual analysis. By leveraging semantic segmentation techniques, our approach enhances detection efficiency while reducing reliance on domain expertise. Furthermore, we systematically evaluate the performance of widely used semantic segmentation models on the landslide detection task. The results demonstrate that models incorporating attention mechanisms achieve superior performance, highlighting their potential for improving landslide identification and segmentation. These findings provide valuable insights into the application of deep learning in landslide monitoring and contribute to the advancement of automated landslide detection methodologies. The intricate topography of landslide-prone regions, coupled with the scarcity of annotated remote sensing data and the inherent limitations of conventional segmentation methods, demands a robust framework capable of adapting to dynamic geomorphological features. To bridge this gap, we present ASK-UNet++, an enhanced semantic segmentation architecture that synergizes multi-scale feature extraction with adaptive attention mechanisms. Our work advances the field through three key innovations:

1. Selective Kernel (SK) attention module: This dynamically recalibrates multi-scale feature weights, resolving ambiguities in complex terrains while preserving fine-grained landslide boundaries.
2. Dual-boundary sliding window strategy: This optimizes data augmentation by retaining critical topographic context and mitigating class imbalance, addressing the challenges of limited training samples.
3. Cross-modal validation: This demonstrates unprecedented generalization capabilities across satellite (Wenchuan) and UAV (Moxi Town) datasets, establishing a versatile tool for real-world landslide monitoring.

Building on these innovations, the paper is structured as follows:

- Section 2 details the CAS Landslide Dataset and preprocessing pipeline, emphasizing the role of our sliding window strategy in enhancing data utility.
- Section 3 elucidates the ASK-UNet++ architecture, with a focus on the SK attention mechanism’s role in feature fusion and its integration with nested skip connections.
- Section 4 rigorously evaluates the model against state-of-the-art benchmarks, validates its generalizability through cross-dataset testing, and quantifies the impact of individual components via ablation studies.
- Section 5 synthesizes the experimental findings, discusses practical implications for disaster risk management, and explores limitations and challenges in real-world deployment.
- Section 6 concludes by summarizing the model’s breakthroughs in accuracy and adaptability, proposes extensions to debris flow detection, and outlines future directions for lightweight real-time deployment and multi-modal data fusion.

## 2. Materials and Methods

### 2.1. Landslide Events in Research Area

**Figure 1:**
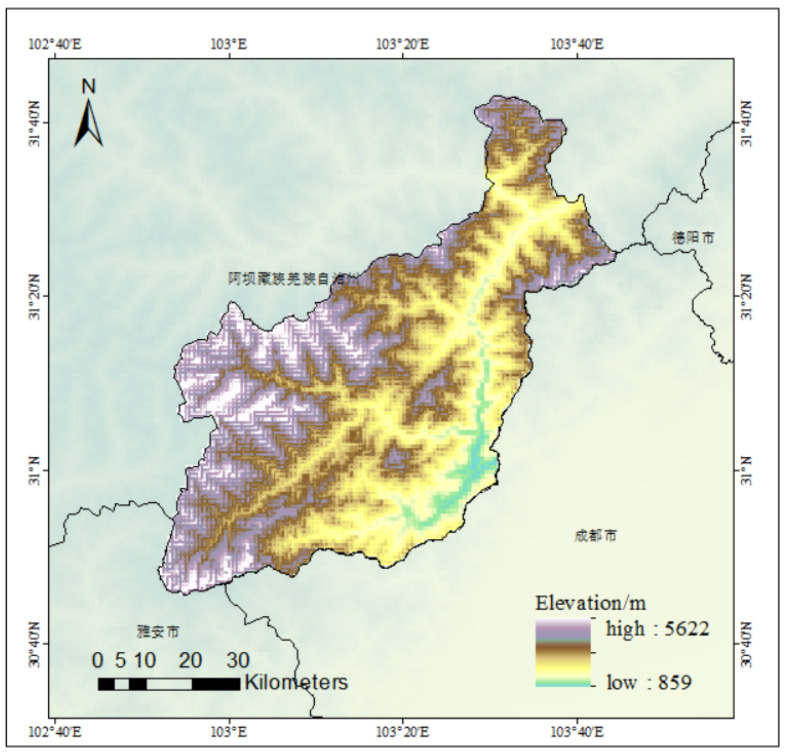
Digital elevation model of Wenchuan.

In 2008, an 8.0-magnitude earthquake struck Wenchuan, causing a large number of landslides. The unique geological structure and topography of the Wenchuan area are inherent factors contributing to landslides. The area is located in the Longmenshan fault zone, with fragmented rocks and complex geological conditions. Long-term crustal movement causes stress concentration inside the mountain and poor stability of the rock and soil mass. Wenchuan has a subtropical monsoon climate with distinct four seasons, characterized by warm and rainy summers and cold, dry winters. The annual precipitation is approximately 500–600 mm, mainly concentrated in summer. As shown in Figure 1, Wenchuan’s terrain is highly variable, with elevations ranging from 859 m to 5622 m. This significant elevation difference leads to pronounced vertical climate variations, resulting in diverse microclimates across different regions. Additionally, the complex topography contributes to frequent hazardous weather events, such as rainstorms and debris flows. As a triggering factor, earthquakes release enormous energy, causing seismic waves to shake the mountain and destroy the original structure of the rock and soil mass, causing it to instantly lose stability. According to research, the higher the peak acceleration of an earthquake, the denser the landslides in the area. At the same time, frequent rainfall after the earthquake in Wenchuan caused rainwater to seep into the ground, increasing the weight of the rock and soil mass, reducing the shear strength, and further triggering landslides. These types of landslides are diverse, including rock landslides, soil landslides, and debris flows formed by landslide transformation [20]. The landslide has brought many difficulties to the post-disaster reconstruction of Wenchuan. The selection of reconstruction sites is fraught with difficulties, and many of the areas originally planned for reconstruction have had to be reassessed and changed due to potential landslide risks. Road construction is repeatedly obstructed, and newly repaired roads are often damaged again due to landslides, resulting in the inability to carry out material transportation and rescue operations smoothly. Water conservation facilities have also been severely affected, with landslides blocking river channels and forming barrier lakes, threatening the safety of downstream residents. The repair and construction of water conservation projects require additional resources for geological reinforcement and hazard investigation. In addition, a large amount of farmland has been buried by landslides, causing severe damage to agricultural production and affecting the livelihoods of local residents, posing more challenges to the reconstruction work.

### 2.2. Dataset and Data Processing

**Figure 2:**
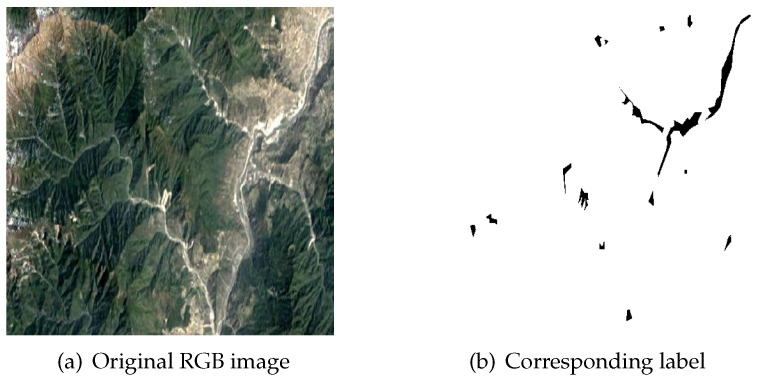
Examples of original RGB image (**a**) and corresponding label (**b**) from the Wenchuan dataset.

As remote sensing technology plays a crucial role in landslide segmentation, we adopted a deep learning-based method to address the challenges inherent in this field. In this study, we utilized subsets from the CAS Landslide Dataset, a novel multi-sensor dataset developed by the Artificial Intelligence Group at the Institute of Mountain Hazards and Environment, Chinese Academy of Sciences (CAS), for deep learning-based landslide detection [21]. The Wenchuan subset served as the training set, while the Moxi-0.2 UAV subset was employed for model transfer learning experiments. Notably, existing public landslide detection datasets are typically limited in scale, ranging from 59 to 3799 samples. In contrast, the CAS Landslide Dataset comprises 20,865 RGB images organized into nine regional subsets, collected through UAV aerial surveys and satellite imagery. Satellite data were acquired from multi-source platforms accessible via Google Earth Engine, while UAV imagery was obtained through collaborative partnerships with precision mapping agencies. For experimental validation, two supplementary datasets were incorporated: the Wenchuan landslide inventory, provided by the USGS, which consists of 178 images captured between November and December 2008, derived from Landsat satellite imagery with a spatial resolution of 5 m; and the high-precision Moxi Town UAV dataset, comprising 1635 images collected between September and October 2022 by the Sichuan Geomatics Centre, with a spatial resolution of 0.2 m. Figure 2 illustrates examples of the original images and corresponding labels from the Wenchuan dataset.

### 2.3. Data Processing

In the original CAS dataset, all images and labels were cropped into 512×512 TIF-format patches, ensuring a one-to-one correspondence between input images and labels for semantic segmentation tasks. However, in this experiment, a different cropping approach is employed. Instead of directly using the predefined patches, the selected training dataset is processed using a sliding window algorithm, where both images and labels are cropped into 224×224 patches.

This approach serves two primary purposes. First, it enables data augmentation, helping to mitigate data imbalance issues. Second, use of the sliding window algorithm allows large images to be decomposed into local contextual windows of 224×224, balancing computational efficiency while preserving key topographical features crucial for landslide detection.

During the cropping process, a dual-boundary adjustment mechanism is implemented to prevent pixel loss. The remaining pixels may be insufficient to form a full-sized window. By adopting this mechanism, when the last sliding window does not fully align with the normal stride, it is right-aligned and bottom-aligned to ensure that all pixels are covered by at least one window.

The sliding window algorithm employs a stride *o* for cropping. Given a window size *S*, the number of sliding steps along the width *W* and height *H* directions is given by

(1) $$N_{h}=\left\lfloor \frac{H-S}{o}\right\rfloor +1,\;N_{w}=\left\lfloor \frac{W-S}{o}\right\rfloor +1$$

If *H* and *W* are not exact multiples of *S*, there may be a remaining region smaller than the window size. To handle this, a dual-boundary adjustment mechanism is applied:

(2) $$p_{h}=\max\left(H-S,0\right),\;p_{w}=\max\left(W-S,0\right)$$

For a given sliding window position (i,j), the top-left coordinates of the window are determined as follows:

(3) $$\left(x_{i},y_{j}\right)=\left(\min\left(i\cdot o,p_{h}\right),\min\left(j\cdot o,p_{w}\right)\right)$$

where $i=0,1,\cdots ,N_{h}-1$ and $j=0,1,\cdots ,N_{w}-1$.

Each cropped sub-image and corresponding label are given by

$$I_{ij}=I\left[x_{i}:x_{i}+S,\;y_{j}:y_{j}+S,\;:\right]$$

$$M_{ij}=M\left[x_{i}:x_{i}+S,\;y_{j}:y_{j}+S\right]$$

**Figure 3:**
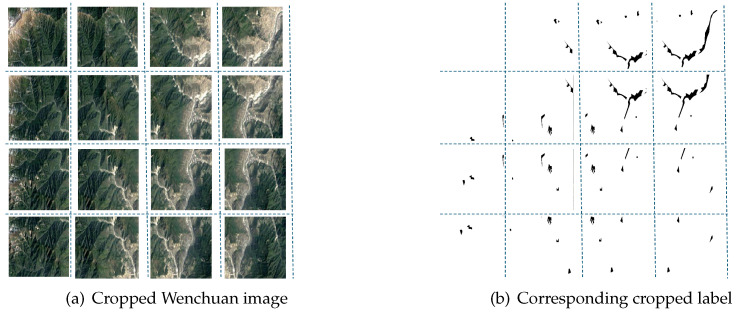
Cropped Wenchuan image (**a**) and corresponding cropped label (**b**).

As shown in Table 2 below, the Wenchuan dataset was expanded from 178 images to 2848 images, while the Moxi Town dataset, acquired using UAV imagery, was expanded from 1635 images to 26,160 images. The cropped Wenchuan images and labels are illustrated in Figure 3 below.

## 3. Methodology

### 3.1. Overall Architecture

**Figure 4:**
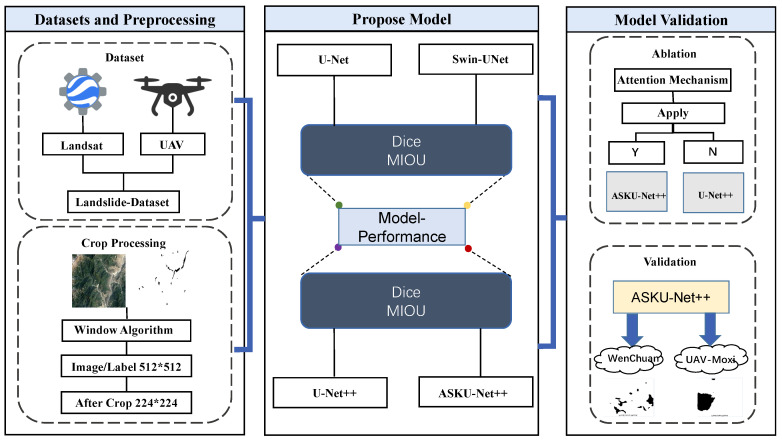
Flowchart of the proposed deep learning model.

Deep learning-based semantic segmentation has demonstrated remarkable success in medical image analysis [22], autonomous driving perception systems, and remote sensing monitoring [23]. Nevertheless, existing models encounter persistent challenges in landslide segmentation, particularly regarding model accuracy and multi-scale feature integration. Conventional architectures including U-Net, Swin-Net, and U-Net++ exhibit limitations in global contextual comprehension, edge refinement capabilities, and parameter optimization efficiency when applied to landslide recognition in complex terrain scenarios. To address these limitations, this paper proposes an innovative deep learning architecture integrating VGG convolutional operations, a U-Net++ framework, and attention mechanisms. The proposed model consists of three principal components working in synergy. A modified VGG backbone network [24] with shallow convolutional layers extracts hierarchical spatial features through its robust local feature representation capacity, establishing fundamental feature maps for subsequent processing. VGG was chosen over other backbones due to its simple yet effective architecture, strong local feature extraction, and balanced computational efficiency. Unlike deeper networks, which rely on complex skip connections, VGG’s sequential convolutional structure preserves fine-grained spatial details. The nested dense skip connection mechanism in U-Net++ facilitates cross-layer feature fusion between encoder and decoder pathways, effectively mitigating the semantic gap inherent in conventional U-Net architectures while enhancing multi-scale feature diversity and representational capacity. Additionally, a Selective Kernel (SK) attention module [25] embedded in the downsampling operations dynamically adjusts weight distributions across multi-scale features, prioritizing critical landslide boundaries and fragmented segmentation regions through adaptive feature recalibration. This hierarchical architecture synergistically combines spatial detail preservation with global context modeling, achieving enhanced performance in handling scale variations and complex geomorphological patterns inherent to landslide recognition tasks. Experimental validation on two datasets acquired through distinct modalities demonstrates that the proposed model achieves enhanced segmentation accuracy in semantic segmentation tasks when compared to baseline models. The experimental design is shown in the flowchart, as detailed in Figure 4. In Figure 4, ASKU-NET++ refers to a Selective Kernel (SK) attention module built upon the U-Net++ architecture.

### 3.2. Model Design

**Figure 5:**
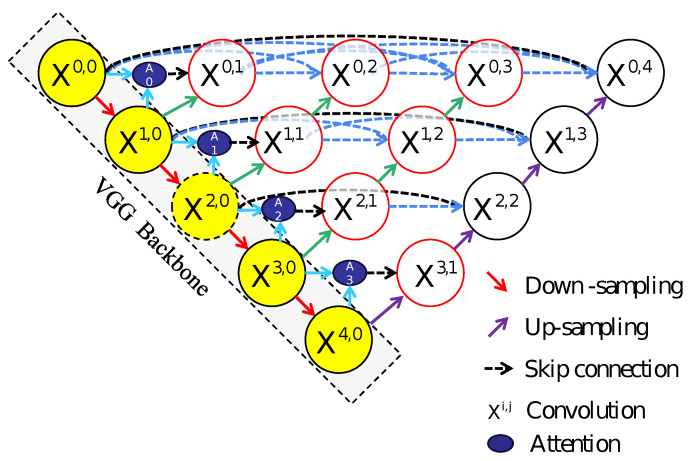
Proposed ASKU-Net++ model.

As illustrated in the design diagrams below Figure 5, the deep learning network is built upon the general framework of U-Net++. Specifically, U-Net++ [26] serves as the backbone for both the downsampling and upsampling layers. U-Net++ is an enhanced version of the original U-Net architecture and is widely adopted for image segmentation tasks. In our design, convolution operations utilize VGG convolutional blocks, given that the shallow convolutional features inherent in VGG are particularly effective for delineating boundaries and segmenting small targets.

As depicted in the flowchart in Figure 4 and the network architecture diagrams in Figure 5, the pre-cropped data and their corresponding labels are fed into the network in a one-to-one mapping. The proposed ASK-UNet++ network is composed of VGG-based convolutional blocks, upsampling modules, and other auxiliary components. Each red arrow indicates a downsampling operation, implemented via max-pooling with a kernel size of 2 and a stride of 2. This procedure halves the spatial dimensions of the input feature maps at each step, thereby reducing the computational load on subsequent convolutional layers and enhancing the network’s overall efficiency. Moreover, this downsampling strategy effectively extracts the essential features from the input while compressing the feature maps, which improves robustness against noise and directs the network’s focus toward more salient structures and features. Consequently, the model’s generalization capability is significantly enhanced.

Conversely, the green arrow denotes the upsampling process, which enlarges the feature maps by a factor of two. Upsampling not only restores the spatial resolution of the feature maps but also preserves a portion of the semantic information contained in the deeper features, thereby progressively recovering the detailed characteristics of the input data. Furthermore, dense skip connections are incorporated between the convolutional blocks to bridge the semantic gap between low-level features and high-level semantic representations, facilitating efficient information transfer across different network layers. This design optimizes gradient flow, alleviates the vanishing gradient problem, and markedly enhances feature reuse, thereby promoting a more comprehensive representation of the input image and bolstering the network’s feature learning and representation capabilities. For instance, consider the $X^{0,4}$ node in Figure 5. This node is directly connected to the preceding $X^{0,3}$ node and is also linked via skip connections to the preceding convolutional units of nodes $X^{0,2}$, $X^{0,1}$, and $X^{0,0}$ within the same layer. Such a multi-scale, multi-path information propagation mechanism enables the integration of features across various scales, thereby promoting the fusion of fine-grained details with high-level semantic information. As a result, this design effectively mitigates the progressive attenuation of information in deep networks, enhances gradient stability, and further improves both feature reuse efficiency and overall learning performance. Assuming that *m* and *n* denote the nodes, and $X^{m,n}$ represents their final feature maps, the computation of $X^{m,n}$ can be defined as follows:

(4) $$X^{m,n}=\left\{\begin{array}{cc} \;H(x^{m-1,n}), & n=0;\\ H\left({\left[x^{m,k}\right]}_{k=0}^{n-1},u\left(x^{m+1,n-1}\right)\right), & n>0. \end{array}\right.$$

**Figure 6:**
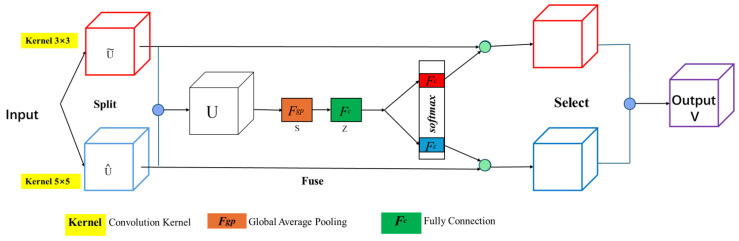
ASK module architecture.

In Equation (4), *m* represents the *m*-th downsampling layer in the encoder of the deep learning architecture, while *n* represents the *n*-th convolutional layer in the skip connection; u(·) corresponds to the upsampling process, which is typically implemented through transposed convolution or interpolation methods to restore low-resolution feature maps to higher spatial dimensions. The process is implemented as H(·), which represents the activation function convolution used to introduce nonlinear feature mappings. The symbol (·) represents the concatenation operation, referring to the concatenation of feature maps from different sources along the channel or spatial dimension. When n=0, the node is an encoder node that only accepts the result from the preceding layer; when n>0, the node accepts (n+1) inputs, including the outputs of the previous *n* nodes from the same layer’s skip connection and the output from the corresponding node of the subsequent upsampling layer (the (m+1)-th layer). This enables the deep fusion of high-level semantic information and fine-grained detail features, improving feature reuse efficiency and overall learning performance, while helping to alleviate the issues of information degradation and vanishing gradients in deep networks. The ASK module employs multi-scale processing by designing parallel branches with varying receptive fields to capture global contextual representations. It utilizes scale-guided softmax attention to compute weight coefficients for each branch, followed by weighted summation of multi-scale features. This mechanism is integrated into VGG convolutional units to adaptively recalibrate feature map weights through spatial attention. The detailed ASK module architecture is shown in Figure 6.

#### 3.2.1. ASK Module

The Attention Selective Kernel mechanism consists of three main components: Split, Fuse, and Select. Its fundamental principle involves designing multi-scale feature extraction layers with varying receptive fields to enhance feature representation. By employing convolution kernels of different sizes, the mechanism adaptively captures multi-scale features and leverages channel-wise attention weights to optimize feature selection.

In the Split stage, the SK module uses convolutional kernels of different sizes to extract input features *X*, generating two feature maps with different scales, $\tilde{U}$ and $\hat{U}$:

(5) $$\tilde{F}:X\to \tilde{U}\in R^{H\times W\times C},\;\hat{F}:X\to \hat{U}\in R^{H\times W\times C}$$

where *H*, *W*, and *C* represent the height, width, and number of channels of the feature map, respectively. In this experiment, 3×3 and 5×5 convolutional kernels are used to generate the feature maps $\tilde{U}$ and $\hat{U}$.

In the Fuse stage, the features $\tilde{U}$ and $\hat{U}$ from different receptive fields are summed to produce the fused feature map *U*:

(6) $$U=\tilde{U}+\hat{U},\;U\in R^{H\times W\times C}$$

A global pooling operation is then applied to *U*, aggregating spatial information for each channel into a single scalar to obtain the feature vector *S*:

(7) $$S_{c}=\frac{1}{H\times W}\sum_{i=1}^{H}\sum_{j=1}^{W}U_{\left(i,j\right)},\;S\in R^{C}$$

After global pooling, the aggregated feature vector *s* is passed through a fully connected transformation to model channel-wise dependencies:

(8) $$Z=\delta \left(\mathrm{BN}\left(W\cdot s+b\right)\right)\in R^{d},\;d=\max\left(\frac{C}{r},L\right)$$

where

- $W\in R^{d\times C}$ is the weight matrix of the fully connected layer;
- $b\in R^{d}$ is the bias vector;
- BN(·) represents batch normalization;
- δ(·) is an activation function;
- *r* is a reduction ratio controlling the bottleneck dimension;
- *L* is a minimum threshold ensuring sufficient feature expressiveness.

In the Select stage, channel-wise attention is applied to adaptively recalibrate feature responses. Given an input feature vector *Z*, two independent fully connected layers compute attention scores for each channel:

(9) $$\begin{array}{cc} a_{c} & =\frac{e^{W_{c}^{a}z}}{e^{W_{c}^{a}z}+e^{W_{c}^{b}z}},\\ b_{c} & =\frac{e^{W_{c}^{b}z}}{e^{W_{c}^{a}z}+e^{W_{c}^{b}z}}, \end{array}\;\forall c\in \left\{1,\cdots ,C\right\}$$

where

- $W_{c}^{a},W_{c}^{b}\in R^{1\times d}$ are the weight parameters of the fully connected layers for each channel;
- $a_{c}$ and $b_{c}$ represent the attention weights for different receptive fields at channel *c*.

The final feature representation is obtained via a weighted sum of the multi-scale features:

(10) $$V=\sum_{c=1}^{C}\left(a_{c}\cdot {\tilde{u}}^{c}+b_{c}\cdot {\hat{u}}^{c}\right)\in R^{H\times W\times C}$$

where $\tilde{U}$ and $\hat{U}$ correspond to the feature maps extracted using different convolutional kernel sizes.

This architecture enables dynamic kernel selection through differentiable attention mechanisms, allowing neurons to adaptively emphasize features from optimal receptive fields based on input characteristics. The experimental implementation demonstrates particular efficacy in multi-scale object recognition tasks, with dual-branch kernel configurations balancing computational efficiency and representational capacity.

#### 3.2.2. Loss Function

In landslide semantic segmentation tasks, the inherent class imbalance between spatially limited landslide regions and extensive background areas often leads to suboptimal performance when employing standard cross-entropy loss functions. To mitigate this problem, we implement the Dice loss function, a metric specifically designed for class-imbalanced scenarios in semantic segmentation.

Dice loss [27] is a commonly used loss function in semantic segmentation, particularly suited for imbalanced class problems. It works by optimizing the model’s ability to maximize the overlap between the predicted and ground truth regions of the landslide, thus reducing the discrepancy between the predictions and the true labels. This approach allows the model to focus more on the smaller landslide class while minimizing the interference from the background during training.

The Dice coefficient, which is used to compute Dice loss, measures the similarity between the predicted and the true labels. The formula for the Dice coefficient is given by

(11) $$\mathrm{Dice}\;\mathrm{Coefficient}=\frac{2\cdot |P\cap G|}{\left|P|+|G\right|}$$

where

- *P* is the set of predicted positive samples.
- *G* is the set of ground truth positive samples.
- |P∩G| represents the number of correctly predicted positive samples.
- |P| denotes the total number of predicted positive samples.
- |G| denotes the total number of actual positive samples.

A higher Dice coefficient indicates greater overlap between prediction and ground truth. To optimize the segmentation models, we define the Dice loss as

(12) DiceLoss=1−DiceCoefficient

**Figure 7:**
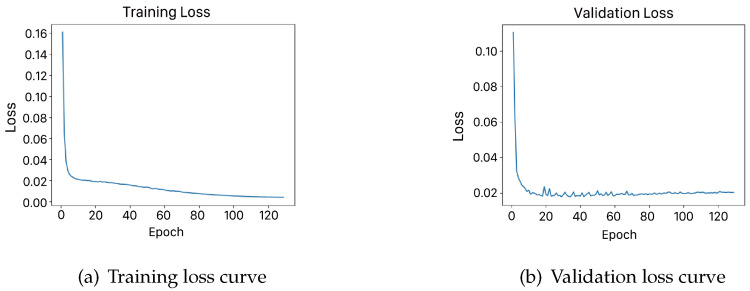
Training and validation loss curves.

This formulation inherently prioritizes accurate delineation of minority-class landslide regions through two key mechanisms. By emphasizing spatial congruence through intersection-over-union optimization rather than pixel-wise classification accuracy, it reduces the disproportionate influence of background pixels during backpropagation. Additionally, the scale-invariant nature of the Dice metric ensures equal weighting of prediction errors regardless of landslide region size, which is particularly crucial for detecting fragmented or small-scale landslide features in remote sensing imagery. When optimized using Dice loss, the formulation effectively mitigates the gradient dominance of background pixels while preserving precise boundary delineation. This makes it particularly suitable for segmenting landslide regions, where variations in shape and scale pose significant challenges in remote sensing applications. To further validate the effectiveness of this method in landslide region segmentation, we optimized the model using Dice loss in this experiment and analyzed its loss variation. The experiments were conducted on an NVIDIA A6000 GPU to ensure efficient training and stable performance. The detailed hardware and software configurations used in the experiment are presented in Table 3. Figure 7 below illustrates the convergence process of the loss function and the final segmentation results.

## 4. Results

This paper proposes an improved U-Net++ model enhanced with the ASK attention mechanism for landslide semantic segmentation. By incorporating a channel–spatial dual-dimensional attention selection mechanism into multi-scale skip connections, the model adaptively focuses on landslide features, thereby improving the segmentation accuracy of deep learning models in landslide segmentation.

### 4.1. Evaluation Metrics

The mean intersection over union (mIoU) is a widely used evaluation metric in semantic segmentation tasks to quantify the region overlap accuracy between predicted results and ground truth labels. Similar to the Dice coefficient, the mIoU evaluates model performance by calculating the ratio of the intersection to union. However, its key distinction lies in computing the intersection over union [28] for each class individually and then averaging across all classes, thereby providing a more comprehensive reflection of the model’s balanced performance in multi-class segmentation.

(13) $$\mathrm{IoU}=\frac{\left|P\cap G\right|}{\left|P\cup G\right|}=\frac{\left|P\cap G\right|}{\left|P|+|G|-|P\cap G\right|}$$

whereas for multi-class segmentation, the mIoU is computed by iterating over all classes:

(14) $$\mathrm{MIoU}=\frac{1}{C}\sum_{i=1}^{C}\frac{|P_{i}\cap G_{i}|}{|P_{i}\left|+\right|G_{i}\left|-\right|P_{i}\cap G_{i}|}$$

where

*C*: Total number of classes;
*P_i_*: Set of pixels predicted as class *i*;
*G_i_*: Set of pixels belonging to class *i* in the ground truth.

While the mean intersection over union (mIoU) offers a detailed evaluation of the model’s performance by measuring the balance between predicted and ground truth labels across all classes, other metrics like F1 score, recall, and overall accuracy are also crucial for understanding different aspects of model performance. These metrics can provide additional insights into a model’s ability to capture the diversity of classes, its sensitivity to true positives, and its general classification accuracy, respectively.

By using a combination of mIoU, F1 score, recall, and overall accuracy, a more nuanced and comprehensive evaluation of a model can be achieved, allowing for a better understanding of its strengths and areas for improvement in multi-class segmentation tasks.

F1 score is a harmonic mean of precision and recall, providing a balanced measure of a model’s accuracy in classification tasks. It is particularly useful when the data are imbalanced. The F1 score is defined as

(15) $$F1=2\cdot \frac{\mathrm{Precision}\cdot \mathrm{Recall}}{\mathrm{Precision}+\mathrm{Recall}\;}$$

where precision and recall are defined as

(16) $$\mathrm{Precision}=\frac{|P_{i}\cap G_{i}|}{|P_{i}|}$$

(17) $$\mathrm{Recall}=\frac{|P_{i}\cap G_{i}|}{|G_{i}|}$$

Recall is a metric that measures the ability of the model to identify all relevant instances of a particular class. It is calculated as the ratio of true positives to the sum of true positives and false negatives:

$$\mathrm{Recall}=\frac{|P_{i}\cap G_{i}|}{|G_{i}|}$$

Overall accuracy quantifies the overall correctness of the model by measuring the proportion of correctly classified pixels to the total number of pixels:

(18) $$\mathrm{Overall}\;\mathrm{Accuracy}=\frac{\sum_{i=1}^{C}\left|P_{i}\cap G_{i}\right|}{\sum_{i=1}^{C}\left|G_{i}\right|}$$

This metric provides an overall assessment of the model’s performance across all classes, ensuring the correctness of the model’s predictions.

### 4.2. Experimental Results

**Figure 8:**
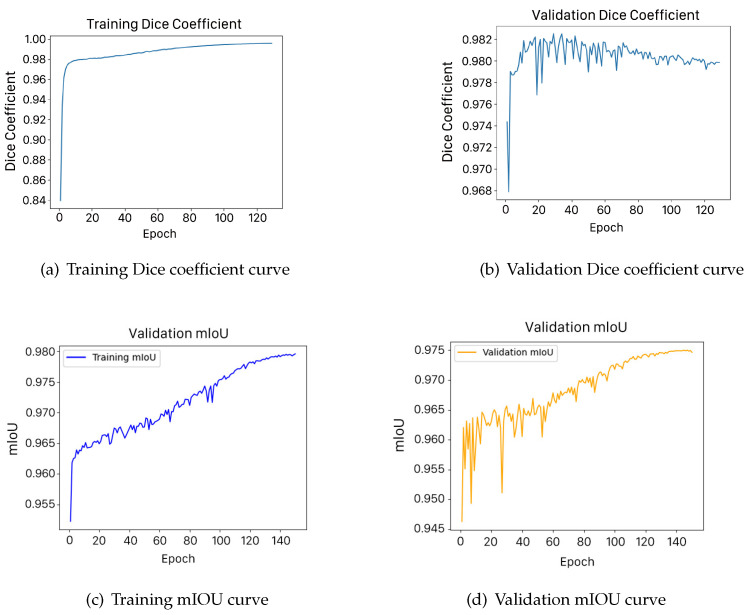
Training and validation curves for Dice coefficient and mIOU.

The experimental results demonstrate that the proposed model achieves breakthrough performance on the public Wenchuan dataset, with the mIoU and Dice scores reaching 97.53% and 98.27%, respectively, outperforming traditional deep learning-based semantic segmentation models. Furthermore, a comparative analysis with baseline models, including U-Net, Swin-UNet, U-Net++, and ASK-UNet++, further validates the superior performance of the proposed model, as shown in Table 4 and Figure 8.

**Figure 9:**
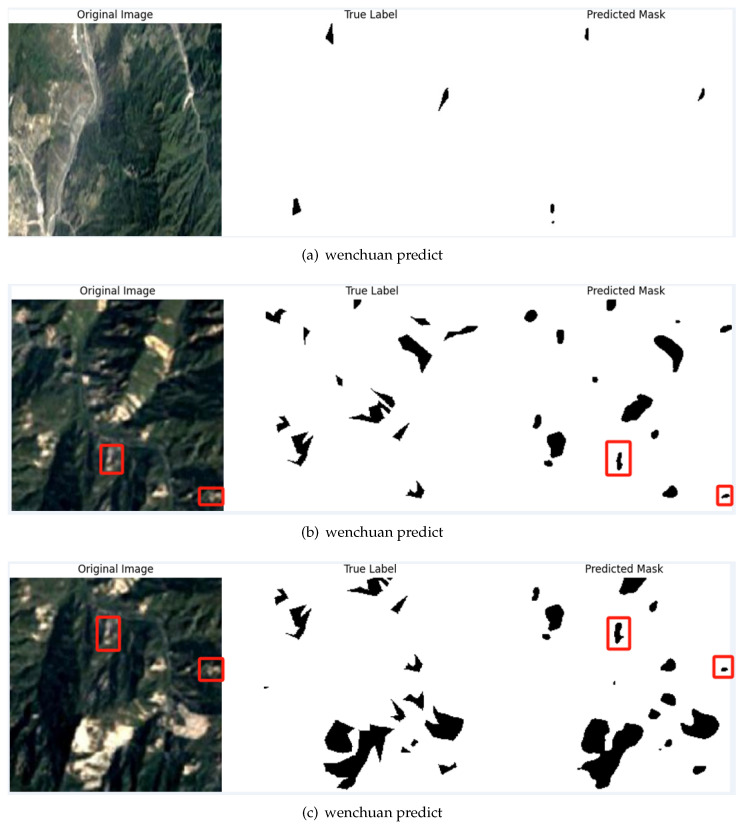
ASKU-Net++ module architecture for landslide prediction on Wenchuan dataset.

As shown in Figure 9, the model’s performance on this dataset further highlights its superior ability in landslide semantic segmentation.

**Figure 10:**
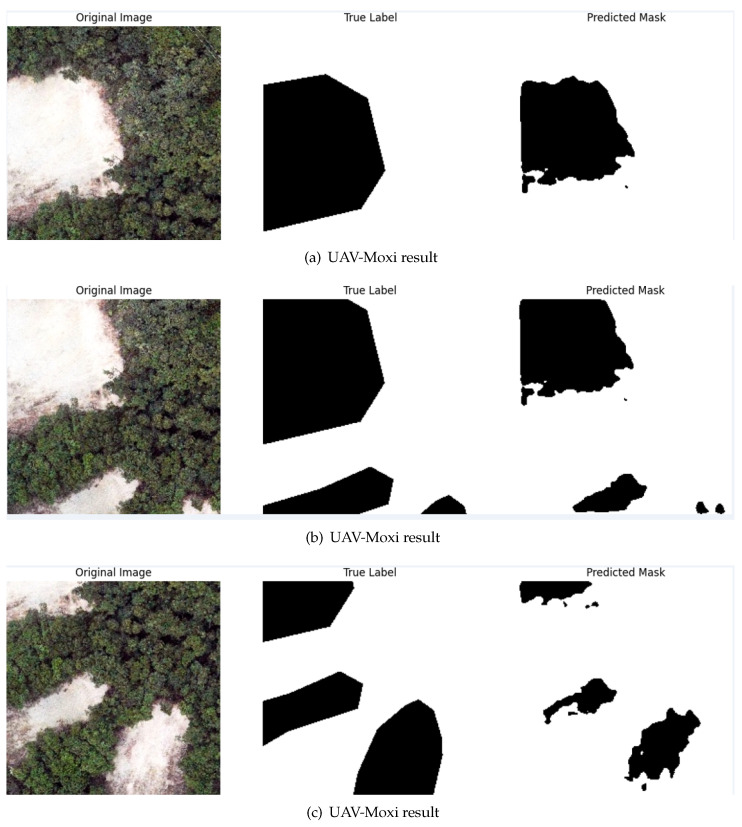
ASKU-Net++ module architecture for landslide prediction on Moxi Town dataset.

After validating the superior performance of the proposed model on the Wenchuan dataset, evaluating the model’s generalization capability, we further evaluated its generalization ability on unseen data. To achieve this, transfer testing was conducted on landslide data collected by UAVs from Moxi Town. The results indicate that the proposed model maintains a segmentation accuracy of 94.6% on landslide samples from Moxi Town, further demonstrating the model’s strong generalization capability on unseen data. The performance of the proposed model on the Moxi Town dataset is shown in Figure 10 below.

## 5. Discussion

The experimental results demonstrate that the proposed model exhibits outstanding semantic segmentation performance on the public Wenchuan dataset, with a mean intersection over union (mIoU) of 97.53% and a Dice coefficient of 98.27%, surpassing conventional deep learning-based semantic segmentation models. As shown in Table 4, comparative analyses with baseline architectures—U-Net, Swin-UNet, and U-Net++—reveal significant improvements, underscoring the efficacy of integrating Selective Kernel (SK) attention mechanisms and multi-scale feature fusion into the U-Net++ framework. The enhanced performance of the proposed ASK-UNet++ model can be attributed to several architectural innovations. First, the SK module dynamically recalibrates feature weights across varying receptive fields, enhancing sensitivity to landslide boundaries and fragmented regions in complex terrain. This adaptive attention mechanism allows the model to focus on the most discriminative features at different scales, which is particularly beneficial for landslide segmentation, where the boundaries between landslide and non-landslide regions can be ambiguous. Second, nested dense skip connections from the U-Net++ architecture facilitate effective feature reuse and gradient flow, enabling more efficient training and better feature representation. This architecture design helps preserve both spatial details and contextual information, which are crucial for accurate segmentation in complex terrain environments. However, the method has certain limitations. The inclusion of SK modules and dense skip connections increases computational complexity, leading to higher memory and processing demands compared to simpler architectures like U-Net. Furthermore, while the sliding window cropping strategy enhances data augmentation, it introduces redundancy in overlapping patches, prolonging preprocessing and inference times. The model’s performance may also be constrained by extreme environmental factors. Despite these computational challenges, the model’s practical utility is evidenced by its robust performance in cross-scenario applications. As illustrated in the visual outputs of the Wenchuan datasets, shown in Figure 9, the model’s segmentation results on the Wenchuan dataset further confirm its ability to delineate landslide boundaries with high precision. Furthermore, the transfer testing results on the Moxi Town UAV dataset in Figure 10 demonstrate the model’s generalization capability across different geographical locations and data acquisition platforms. Despite the differences in image resolution, perspective, and environmental conditions between satellite imagery (Wenchuan) and UAV imagery (Moxi Town), the model maintains a high segmentation accuracy of 94.6%. This cross-scenario adaptability suggests that the proposed approach could be deployed in various landslide monitoring applications without extensive retraining. Beyond surpassing baseline architectures, our model shows unique advantages in comparison to specialized landslide segmentation frameworks. For instance, Le et al. [29] proposed an enhanced U-Net model that incorporated residual convolution layers and multi-head attention, achieving an mIoU of 76.07% and an F1 score of 84.07% on the Landslide4Sense dataset. Soares et al. [30] examined model generalization in rainfall-induced landslides in Brazil, finding that model performance was significantly affected by patch size and environmental variability, and achieved lower mIoU values despite post-processing efforts. In contrast, our ASK-UNet++ maintains high accuracy and generalization performance across diverse conditions. Furthermore, the Landslide4Sense benchmark study by Ghorbanzadeh et al. [31] evaluated several deep learning models and identified ResU-Net as the top performer. However, its mIoU performance (approx. 76%) remains notably below that of the proposed ASK-UNet++. In contrast, our ASK-UNet++ model demonstrates strong cross-scenario adaptability across multiple datasets, achieving stable, high-precision segmentation in various geographical regions and data collection methods. This highlights the generalizability of our model in landslide segmentation tasks, particularly in handling complex terrain and ambiguous boundaries.

## 6. Conclusions

This study introduces a novel approach by integrating a Selective Kernel (SK) attention module with a nested dense skip connection architecture based on the U-Net++ framework. This design effectively addresses the class imbalance issue in landslide segmentation. The SK module adaptively recalibrates feature weights through multi-scale kernel selection, enabling precise focus on landslide boundaries and fragmented regions. Additionally, the nested dense skip connections in U-Net++ enhance multi-scale feature fusion, preserving spatial details and contextual information to improve segmentation accuracy. The experimental results demonstrate the superiority of ASKU-Net++ in landslide segmentation tasks. On the Wenchuan dataset, our model achieves a 97.53% mIoU and 98.27% Dice coefficient, surpassing baseline models including U-Net (92.73% mIoU), Swin-UNet (91.27% mIoU), and standard U-Net++ (94.25% mIoU) by significant margins. The 96.04% overall accuracy further highlights its balanced performance across landslide and non-landslide regions. To validate cross-scenario adaptability, we conducted transfer learning experiments on the Moxi Town UAV dataset. Despite differences in sensor modalities and geographical conditions, ASKU-Net++ maintained 94.6% accuracy, demonstrating robust generalization capabilities for diverse landslide detection scenarios. However, several limitations and challenges warrant consideration. The model’s performance remains dependent on the quality and quantity of annotated training data, where limited or biased datasets (e.g., under-represented landslide types or regions) may reduce accuracy. While the nested skip connections and SK attention modules improve feature fusion, they increase computational demands during training. Additionally, variations in sensor resolution (e.g., drone vs. satellite imagery) and environmental conditions may introduce segmentation uncertainties. Manual annotation subjectivity, particularly for fragmented or small-scale features, could also propagate noise into the training process. Future research should focus on (1) enhancing robustness through semi-supervised learning to reduce annotation costs, (2) optimizing computational efficiency for real-time applications, and (3) integrating multi-modal data (e.g., LiDAR, InSAR) to mitigate sensor-specific uncertainties. Furthermore, the modular design proposed in this paper makes the method not only applicable to landslide segmentation but also extendable to the intelligent identification of other geological disasters, such as debris flows, broadening its potential application. The flexibility of this design allows the system to adapt to different disaster types, providing a new technical pathway for future disaster monitoring and early warning systems. In comparison with traditional methods, deep learning demonstrates significant advantages in landslide detection and segmentation, particularly in processing high-resolution remote sensing imagery. Deep learning showcases greater accuracy, robustness, and operational efficiency, providing strong technical support for the intelligent detection of geological disasters. Despite existing challenges, ASKU-Net++’s validated accuracy and modular architecture pave the way for broader applications in geological hazard management and risk mitigation, advancing the field toward more efficient and intelligent solutions.

## Figures and Tables

**Table 1:** Comparative analysis of research methods for landslide assessment.

Research Method	Advantages	Limitations
Ground Investigation [14,15]	Provides high-resolution, first-hand data through direct observation.	Time-intensive and labor-intensive, with limited spatial coverage.
	Enables in situ assessment of landslide characteristics.	Potential safety hazards in unstable or inaccessible terrains.
	Facilitates engagement with local communities to obtain historical insights.	Highly dependent on investigator expertise, introducing subjectivity.
	Allows for precise evaluation of lithological and geotechnical properties.	Logistically challenging in remote or hazardous locations.
	Provides physical subsurface samples for comprehensive analysis.	High operational costs due to drilling and laboratory analysis.
Geological Exploration [16]	Reveals concealed geological structures beneath the surface.	Requires specialized equipment and technical expertise.
	Offers reliable quantitative data for landslide stability assessment.	Potential environmental disruption, necessitating site rehabilitation.
	Enables large-scale and efficient data acquisition.	Optical sensors are susceptible to atmospheric conditions and cloud cover.
Remote Sensing Techniques [17]	Facilitates multi-temporal monitoring for dynamic change detection.	Limited capability for analyzing subsurface geological conditions.
	Allows access to hazardous or remote regions without physical presence.	Requires advanced data processing techniques and expertise.
Radar Data [18]	Supports long-term monitoring of terrain deformation.	Geometric distortions are prevalent in steep or rugged terrains.
	Enables large-scale assessment of multiple hazard-prone areas.	Susceptible to atmospheric interference and spatiotemporal decorrelation.
	Operates independently of weather conditions, ensuring consistent observations.	Phase unwrapping complexities may introduce errors in analysis.
	Captures surface displacement patterns with high temporal resolution.	Atmospheric noise can compromise measurement accuracy.
	Detects small-scale deformations with high precision.	Data processing and interpretation are computationally intensive.
		Inter-platform variability hinders seamless data integration.

**Table 2:** Comparison of original and augmented dataset sizes.

Dataset	Date	Resolution	Original Image Amount	After Crop Image Amount	Original Label Amount	After Crop Label Amount
Wenchuan	Nov–Dec 2008	5 m	178	2848	178	2848
Moxi Town	Sep–Oct 2022	0.2 m	1635	26,160	1635	26,160

**Table 3:** Experimental configuration.

Parameter	Value
Initial learning rate	$1\times 10^{-3}$
Batch size	16
Number of epochs	120–150
Optimizer	Adam
Learning rate scheduler	Cosine Annealing
Early stopping patience	150
Validation	20%
Test set	20%
Training set	60%
Regularization	L2 Regularization (weight decay), Dropout (0.5)
Hardware environment	NVIDIA RTX A6000 (NVIDIA, Santa Clara, CA, USA)

**Table 4:** Comparison of segmentation performance on the Wenchuan dataset.

Model	mIoU (%)	Dice (%)	F1 (%)	Precision (%)	Recall (%)	Accuracy (%)
U-Net	92.73	91.43	93.28	90.12	91.56	92.45
Swin-UNet	91.27	92.36	90.89	92.15	91.78	93.51
U-Net++	94.25	93.81	93.17	94.59	92.88	95.23
ASK-UNet++	97.53	98.27	97.53	98.40	96.76	96.04

## Data Availability

The data used in this paper are publicly available and can be accessed via the GitHub repository: https://github.com/Aizu0/CAS-Landslide-Dataset-production-code.git (accessed on 20 April 2025). The dataset is developed and maintained by the Artificial Intelligence Group at the Institute of Mountain Hazards and Environment, Chinese Academy of Sciences.

## References

[1] Highland L.M., Bobrowsky P. (2008). The Landslide Handbook—A Guide to Understanding Landslides.

[2] Hungr O., Leroueil S., Picarelli L. (2014). The Varnes classification of landslide types, an update. Landslides.

[3] Hou T.S., Xu G.L., Shen Y.J., Wu Z.Z., Zhang N.N., Wang R. (2013). Formation mechanism and stability analysis of the Houba expansive soil landslide. Eng. Geol..

[4] Ni H., Tang C., Zheng W., Xu R., Tian K., Xu W. (2014). An overview of formation mechanism and disaster characteristics of post-seismic debris flows triggered by subsequent rainstorms in Wenchuan earthquake extremely stricken areas. Acta Geol. Sin.-Engl. Ed..

[5] Batar A.K., Watanabe T. (2021). Landslide susceptibility mapping and assessment using geospatial platforms and weights of evidence (WoE) method in the Indian Himalayan Region: Recent developments, gaps, and future directions. ISPRS Int. J. Geo-Inf..

[6] Kahlon S., Chandel V.B., Brar K.K. (2014). Landslides in Himalayan mountains: A study of Himachal Pradesh, India. Int. J. IT Eng. Appl. Sci. Res..

[7] Jibson R.W., Harp E.L., Michael J.A. (2000). A method for producing digital probabilistic seismic landslide hazard maps. Eng. Geol..

[8] Cruden D.M., Varnes D.J. (1996). Landslide types and processes. Landslides: Investigation and Mitigation.

[9] Pazzi V., Morelli S., Fanti R. (2019). A review of the advantages and limitations of geophysical investigations in landslide studies. Int. J. Geophys..

[10] Jensen J.R. (2009). Remote Sensing of the Environment: An Earth Resource Perspective 2/e.

[11] Ghaderpour E., Antonielli B., Bozzano F., Mugnozza G.S., Mazzanti P. (2024). A fast and robust method for detecting trend turning points in InSAR displacement time series. Comput. Geosci..

[12] Kalavrezou I.E., Castro-Melgar I., Nika D., Gatsios T., Lalechos S., Parcharidis I. (2024). Application of time series INSAR (SBAS) method using sentinel-1 for monitoring ground deformation of the Aegina Island (Western Edge of Hellenic Volcanic Arc). Land.

[13] Cheng Y., Pang H., Li Y., Fan L., Wei S., Yuan Z., Fang Y. (2025). Applications and Advancements of Spaceborne InSAR in Landslide Monitoring and Susceptibility Mapping: A Systematic Review. Remote Sens..

[14] Chambers J., Wilkinson P., Kuras O., Ford J., Gunn D., Meldrum P., Pennington C., Weller A., Hobbs P., Ogilvy R. (2011). Three-dimensional geophysical anatomy of an active landslide in Lias Group mudrocks, Cleveland Basin, UK. Geomorphology.

[15] Sujitapan C., Kendall J., Chambers J., Yordkayhun S. (2024). Landslide assessment through integrated geoelectrical and seismic methods: A case study in Thungsong site, southern Thailand. Heliyon.

[16] Superczyńska M., Maślakowski M., Mieszkowski R. (2024). Three-dimensional interpretation of geophysical and geotechnical investigation of landslides. Arch. Civ. Eng..

[17] Darvishi M., Schlögel R., Kofler C., Cuozzo G., Rutzinger M., Zieher T., Toschi I., Remondino F., Mejia-Aguilar A., Thiebes B. (2018). Sentinel-1 and ground-based sensors for continuous monitoring of the Corvara Landslide (South Tyrol, Italy). Remote Sens..

[18] Jiang Y., Liao M., Zhou Z., Shi X., Zhang L., Balz T. (2016). Landslide deformation analysis by coupling deformation time series from SAR data with hydrological factors through data assimilation. Remote Sens..

[19] Zhang Q., Wang T. (2024). Deep learning for exploring landslides with remote sensing and geo-environmental data: Frameworks, progress, challenges, and opportunities. Remote Sens..

[20] Fan X., Scaringi G., Domènech G., Yang F., Guo X., Dai L., He C., Xu Q., Huang R. (2019). Two multi-temporal datasets that track the enhanced landsliding after the 2008 Wenchuan earthquake. Earth Syst. Sci. Data.

[21] Xu Y., Ouyang C., Xu Q., Wang D., Zhao B., Luo Y. (2024). CAS Landslide Dataset: A Large-Scale and Multisensor Dataset for Deep Learning-Based Landslide Detection. Sci. Data.

[22] Ronneberger O., Fischer P., Brox T. (2015). U-net: Convolutional networks for biomedical image segmentation. Proceedings of the Medical Image Computing and Computer-Assisted Intervention–MICCAI 2015: 18th International Conference.

[23] Yuan Q., Shen H., Li T., Li Z., Li S., Jiang Y., Xu H., Tan W., Yang Q., Wang J. (2020). Deep learning in environmental remote sensing: Achievements and challenges. Remote Sens. Environ..

[24] Simonyan K., Zisserman A. Very Deep Convolutional Networks for Large-Scale Image Recognition. Proceedings of the International Conference on Learning Representations.

[25] Li X., Wang W., Hu X., Yang J. Selective kernel networks. Proceedings of the IEEE/CVF Conference on Computer Vision and Pattern Recognition.

[26] Zhou Z., Rahman Siddiquee M.M., Tajbakhsh N., Liang J. (2018). Unet++: A nested u-net architecture for medical image segmentation. Proceedings of the Deep Learning in Medical Image Analysis and Multimodal Learning for Clinical Decision Support: 4th International Workshop, DLMIA 2018, and 8th International Workshop, ML-CDS 2018.

[27] Jadon S. (2020). A survey of loss functions for semantic segmentation. Proceedings of the 2020 IEEE Conference on Computational Intelligence in Bioinformatics and Computational Biology (CIBCB).

[28] Everingham M., Van Gool L., Williams C.K., Winn J., Zisserman A. (2010). The pascal visual object classes (voc) challenge. Int. J. Comput. Vis..

[29] Le C., Pham L., Lampert J., Schlögl M., Schindler A. (2024). Landslide Detection and Segmentation Using Remote Sensing Images and Deep Neural Networks. Proceedings of the IGARSS 2024–2024 IEEE International Geoscience and Remote Sensing Symposium.

[30] Soares L.P., Dias H.C., Garcia G.P.B., Grohmann C.H. (2022). Landslide segmentation with deep learning: Evaluating model generalization in rainfall-induced landslides in Brazil. Remote Sens..

[31] Ghorbanzadeh O., Xu Y., Ghamisi P., Kopp M., Kreil D. (2022). Landslide4sense: Reference benchmark data and deep learning models for landslide detection. arXiv.

